# Synthesis and Characterization of the CaTiO_3_:Eu^3+^ Red Phosphor by an Optimized Microwave-Assisted Sintering Process

**DOI:** 10.3390/ma13040874

**Published:** 2020-02-15

**Authors:** Haifeng Wang, Jianwei Lu, Ruoxuan Wang, Yungu Dong, Linfeng Ding

**Affiliations:** 1 College of Materials Science and Engineering, Donghua University, Shanghai 201620, China; 2 Engineering Research Center of Advanced Glass Manufacturing Technology, Ministry of Education, Donghua University, Shanghai 201620, China

**Keywords:** CaTiO_3_:Eu^3+^ red phosphors, microwave-assisted sintering, chemical co-precipitation method, luminescent properties

## Abstract

The synthesis process has a significant influence on the properties of Ca_1-x_TiO_3_:Eu^3+^_x_ phosphors; thus, an optimized process will lead to a better performance of the Ca_1-x_TiO_3_:Eu^3+^_x_ phosphors. In this work, the feasibility of synthesizing the Ca_1-x_TiO_3_:Eu^3+^_x_ phosphor with a good luminescent performance by combining the chemical co-precipitation method and microwave-assisted sintering was studied. The precursor of Ca_1-x_TiO_3_:Eu^3+^_x_ phosphors were prepared by the chemical co-precipitation method. To find an optimized process, we applied both of the traditional (furnace) sintering and the microwave-assisted sintering to synthesize the Ca_1-x_TiO_3_:Eu^3+^_x_ phosphors. We found out that a sintering power of 528 W for 50 min (temperature around 950 °C) by a microwave oven resulted in similar emission intensity results compared to traditional furnace sintering at 900 °C for 2.5 h. The synthesized Ca_1-x_TiO_3_:Eu^3+^_x_ phosphors has an emission peak at 617 nm (^5^D_0_→^7^F_2_), which corresponds to the red light band. This new synthesized method is an energy efficient, time saving, and environmentally friendly means for the preparation of Ca_1-x_TiO_3_:Eu^3+^_x_ red phosphor with good luminescent performance.

## 1. Introduction

Calcium titanate (CaTiO_3_) represents one of the most important classes of mixed oxides owing to its good chemical and thermal stability as well as great mechanical resistance. Particularly, rare earth–activated CaTiO_3_ phosphors [1,2,3,4] exhibit high-efficiency luminescent properties and, therefore, are promising for using in various optoelectronic devices, e.g., field-emission displays and long persistent phosphorescent phosphors. Such phosphors are crucial for applications in white light–emitting diodes to adjust their chromogenic performance, color and temperature [5].

Eu^3+^ ions have significant importance due to their potential application as red phosphors, electroluminescent devices, optical amplifiers, and lasers when it is used as doping in a great variety of materials [6]. To date, the perovskite phosphors can be prepared by various methods [1,2,3,4,5,6,7,8], including high-temperature solid-phase sintering, the sol-gel method, the chemical coprecipitation method, a hydrothermal process, the combustion method, etc. Studies [1,2,3,4,5,6,7,8] have shown that the preparation process has a significant influence on the properties of Ca_1-x_TiO_3_:Eu^3+^_x_ phosphors; thus, an optimized process will lead to a better performance of the Ca_1-x_TiO_3_:Eu^3+^_x_ phosphors.

The chemical co-precipitation method [9,10] to prepare Ca_1-x_TiO_3_:Eu^3+^_x_ phosphors is processed by dissolving the calcium carbonate (CaCO_3_) in the acetic acid and then mixing the solution with the europium acetate and the titanium dioxide by stirring for the precipitation. The precipitation is washed several times, which is followed by a traditional sintering (in a muffle furnace) to obtain the Ca_1-x_TiO_3_:Eu^3+^_x_ phosphors powder.

In recent years, the microwave-assisted sintering method [2,11,12,13,14] has been widely applied in the synthesis of phosphor and ceramic materials due to its advantages of rapid heating rate and energy efficiency [15]. Different from the conventional sintering method, with radiation or conduction from the surface to the core as the main heat transfer mechanism, the microwave-assisted sintering procedure heats the whole sample by volumetric heating from microwaves [12]. Thus, to combine advantages from the chemical co-precipitation method and the microwave-assisted sintering method will be an optimized process (e.g., time and energy saving) to prepare Ca_1-x_TiO_3_:Eu^3+^_x_ phosphors with better performance.

In this work, the precursor of Ca_1-x_TiO_3_:Eu^3+^_x_ phosphor was synthesized by the chemical co-precipitation method. Then, we applied both the traditional sintering and the microwave-assisted sintering to prepare the Ca_1-x_TiO_3_:Eu^3+^_x_ phosphor. The luminescence properties from the optimized process were studied and compared to the traditional method.

## 2. Experimental Procedures

### 2.1. Sample Preparation

Main raw materials including Ca(NO_3_)_2_·4H_2_O, Eu(NO_3_)_3_·6H_2_O, C_2_H_2_O_4_·2H_2_O, (NH_4_)_2_C_2_O_4_, and C_16_H_36_O_4_Ti with a purity of 99.9% (Sinopharm Group, Shanghai, China) were used as starting materials.

**Figure 1:**
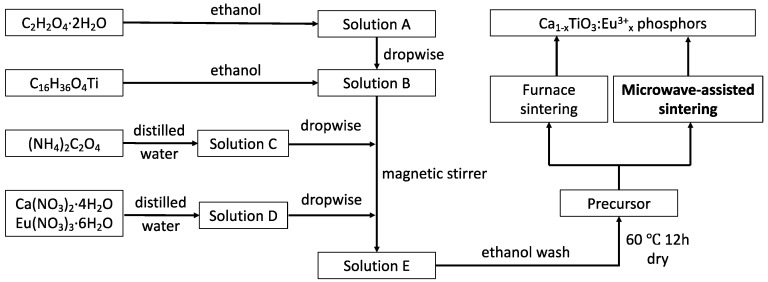
Flow chart of the experimental process for synthesizing the Ca_1-x_TiO_3_:Eu^3+^_x_ phosphors.

The sample synthesizing process is presented in Figure 1. Firstly, we applied the chemical co-precipitation method to synthesize the precursor. The C_2_H_2_O_4_·2H_2_O and C_16_H_36_O_4_Ti were dissolved in the ethanol to get solutions A and B. The (NH_4_)_2_C_2_O_4_ was dissolved in the distilled water to obtain a solution C. The Ca(NO_3_)_2_·4H_2_O and Eu(NO_3_)_3_·6H_2_O were dissolved in the distilled water to get solution D. Solution A and solution C were dropwise added to solution B and magnetic stirred to obtain a clear solution E, followed by dropwise adding solution D to solution E and magnetic stirring for 2 h. The precipitation was centrifuged washing and dried to get the precursor. Then, the precursors were transferred into alumina crucibles and heated in a muffle furnace at 600–900 °C for 2.5 h in air as well as heated in a microwave oven (Galanz, G80F 2.45G Hz) at 528–800 W for 10–60 min to prepare the Ca_1-x_TiO_3_:Eu^3+^_x_ phosphors. During the microwave assisted sintering, a small crucible with dry Ca_0.97_TiO_3_: Eu^3+^_0.03_ phosphor powder was placed in a large crucible filled with 3/4 volume of activated carbon. The precursor was heated by heat transfer from the activated carbon to prepare the Ca_0.97_TiO_3_: Eu^3+^_0.03_ phosphor.

### 2.2. Characterization Techniques

The chemical structure of the precursor was measured by applying Fourier transform infrared spectroscopy (FT-IR, Nexus-670, Waltham, MA, USA) measurements, where the precursor samples were mounted in the KBr pelletized disks and measured in the 400–3600 cm^−1^ region with a resolution of 2 cm^−1^. The thermal properties of the precursor were tested using a simultaneous thermal analysis (TG-DSC, STA409PC, Selb, Germany) in air atmosphere. The crystal information was analyzed by an X-ray diffractometer (XRD, D/max-2500PC, Rigaku, Japan) in air atmosphere. The micromorphology of the samples was studied by using field emission scanning electron microscope (SEM, S-3000N, Tokyo, Japan). The luminescent properties of the phosphors were tested by the fluorescence spectroscopy (FP-6600, Tokyo, Japan).

## 3. Results and Discussion

### 3.1. Precursor Analysis

**Figure 2:**
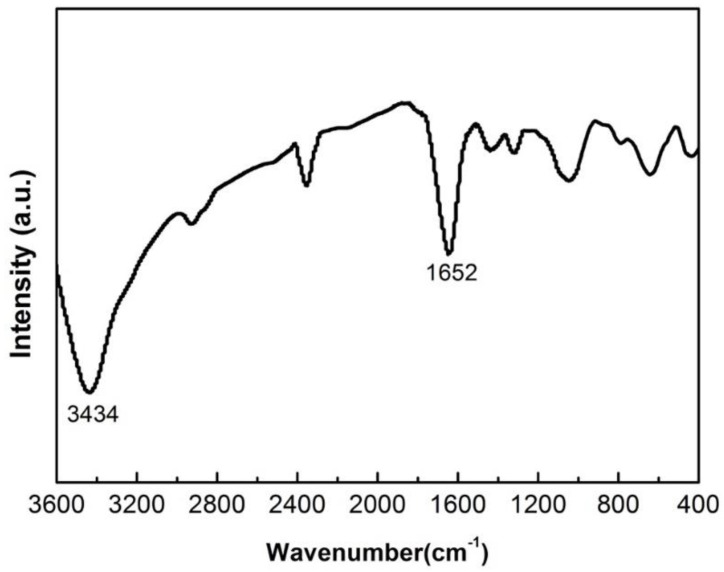
The FT-IR plot of the precursor from the chemical co-precipitation method.

In order to understand the structure of precursor (from the chemical co-precipitation method [9,10]) and carry out subsequent processing, the precursor was tested by FT-IR, and the results are shown in the Figure 2. From the FT-IR curve, the peak at around 3434 cm^−1^ is the anti-symmetry and telescopic symmetrical vibration of OH, which corresponds to the H-O-H group of water bound in the precursor. The peak around 1600 cm^−1^ is the vibration of C=O, which corresponds to the carbon–oxygen double bond inside the MTiO(C_2_O_4_)·4H_2_O. The metal cations and complexes of TiO(C_2_O_4_)^2−^ are uniformly represented by MTiO(C_2_O_4_) [16].

**Figure 3:**
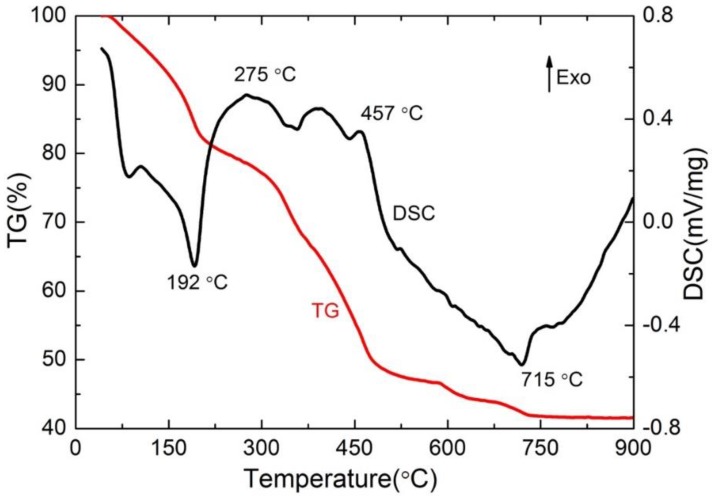
The TG-DSC curve of the precursor.

To optimize the sintering process, the understanding of the thermal properties of the precursor is crucial. Figure 3 shows the thermal decomposition of the precursor. The TG curve has a clear three-stage decline during the heating process which is corresponding to the three endothermic peaks in the DSC curve [17,18,19]. At the temperature before 200 °C (stage one), the precursor first lost the free water and then lost the bound water. The endothermic peak around 200 °C in the DSC curve indicates the loss of bound water. The reaction [17] can be represented by Equation (1).

MTiO(C_2_O_4_)·4H_2_O→MTiO(C_2_O_4_)_2_ + 4H_2_O (1)

The second stage at the temperature range of 275−457 °C, where the precursor decomposed to MCO_3_ and TiO_2_ by releasing the CO and CO_2_. The process companies by an obvious weight loss [17] which can be represented by Equation (2).

MTiO(C_2_O_4_)_2_→MCO_3_ + TiO_2_ + CO + CO_2_ (2)

The third stage mainly occurred in solid-phase reaction at 500–900 °C. The reaction process [17] is shown in Equation (3).

MCO_3_ + TiO_2_→MTiO_3_ + CO_2_ (3)

The TG curve is getting flat when the temperature is higher than 715 °C, which indicates that the reaction was completed.

### 3.2. Ca_1-x_TiO_3_:Eu^3+^_x_ Phosphor Synthesized by the Traditional Sintering Processes

Based on the TG-DSC curve (Figure 3), the MTiO_3_ will be generated at a temperature higher than 600 °C. Thus, the effect of sintering temperature on crystal formation and luminescence properties are firstly explored.

**Figure 4:**
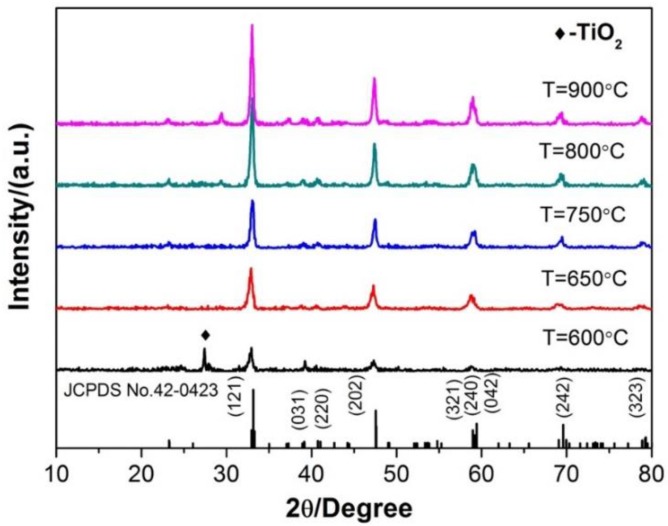
XRD patterns of the Ca_1-x_TiO_3_:Eu^3+^_x_ phosphor samples at different traditional sintering temperatures and same isothermal holding for 2.5 h.

Figure 4 shows the XRD patterns of the Ca_1-x_TiO_3_:Eu^3+^_x_ phosphor samples at different sintering temperatures and the same isothermal holding for 2.5 h. It can be seen from Figure 4 that the Ca_1-x_TiO_3_:Eu^3+^_x_ has been synthesized at 600 °C while contains a certain amount of TiO_2_ [20,21]. When the sintering temperature is higher than 650 °C, the pure Ca_1-x_TiO_3_:Eu^3+^_x_ phosphor can be produced (without TiO_2_ crystal peak). The diffraction peak (diffraction angle: 33.24°, 47.74°, 59.54°) corresponds to the standard card JCPDS NO.42-0423, which is the orthorhombic CaTiO_3_ structure. Moreover, the intensity of the standard diffraction peak is getting stronger with increasing the sintering temperature. The intensity of the standard diffraction peak is greatly enhanced at sintering temperature higher than 800 °C, which indicates good crystallization.

**Figure 5:**
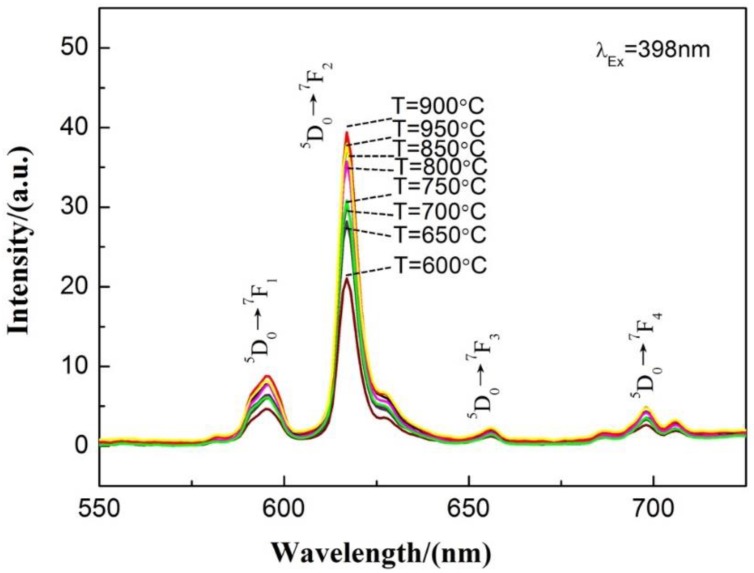
Emission spectra under an excitation wavelength of 398 nm of the Ca_0.97_TiO_3_:Eu^3+^_0.03_ prepared at different traditional sintering temperature.

Figure 5 shows the emission spectra under an excitation wavelength of 398 nm of Ca_0.97_TiO_3_:Eu^3+^_0.03_ prepared at different traditional sintering temperature. It can be seen from Figure 5 that the luminous performance of the Ca_0.97_TiO_3_:Eu^3+^_0.03_ powder is continuously increasing with increasing of sintering temperature. The overall trend of luminescent properties is consistent with the trend of the crystal structure presented by XRD. In other words, the crystallization is not complete at relatively lower temperatures under 800 °C. The incomplete crystallization will result in weak light intensity.

Based on the results from the XRD and the luminescence properties, a traditional sintering temperature of 900 °C is the best process for preparing the Ca_0.97_TiO_3_:Eu^3+^_0.03_ phosphor.

**Figure 6:**
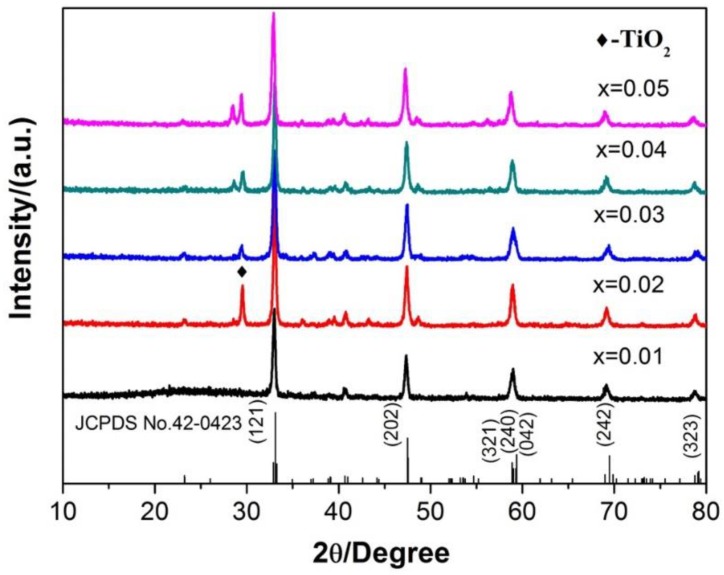
XRD patterns of the Ca_1-x_TiO_3_:Eu^3+^_x_ phosphors doped with various concentrations of Eu^3+^ synthesized by the traditional sintering processes.

To determine the best concentration of Eu^3+^ in the Ca_1-x_TiO_3_:Eu^3+^_x_ phosphor, the Ca_1-x_TiO_3_:Eu^3+^_x_ phosphors doped with various concentrations of Eu^3+^ are synthesized. The XRD patterns of the Ca_1-x_TiO_3_:Eu^3+^_x_ phosphors doped with various concentrations of Eu^3+^ are shown in Figure 6. The main diffraction peak (diffraction angle: 33.24°, 47.74°, 59.54°) is consistent with the standard card JCPDS NO.42-0423, which is the orthorhombic CaTiO_3_ structure. The TiO_2_ peaks are also detected in the XRD pattern, which is due to the increase of Eu^3+^ accompanied by an excessive adding of Ti to ensure the complete chemical reaction of the metal cation (Ca^2+^, Eu^3+^) and [TiO(C_2_O_4_)_2_]^2−^. Table 1 shows the lattice constant of the Ca_1-x_TiO_3_:Eu^3+^_x_ phosphors calculated by JADE software (6.5). It can be seen in Table 1 that the lattice constant decreases slightly with the addition of Eu^3+^ content, which is as a reason for the distance decrease of the lattice and the surrounding O^2−^ ion. Indeed, the ion radius (94.7 pm) of Eu^3+^ is slightly smaller than the ion radius (100 pm) of Ca^2+^. Moreover, the change of lattice constant also proved that the Eu^3+^ ions are successfully doped into the crystal structure.

**Figure 7:**
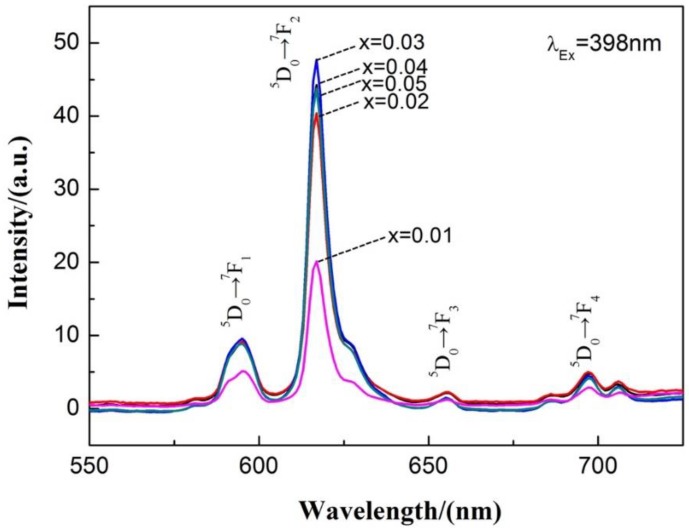
Emission spectra under an excitation wavelength of 398 nm of the Ca_1-x_TiO_3_:Eu^3+^_x_ phosphors doped with various concentrations of Eu^3+^ synthesized by the traditional sintering processes.

Figure 7 shows the emission spectra under an excitation wavelength of 398 nm of Ca_1-x_TiO_3_:Eu^3+^_x_ phosphors doped with various concentrations of Eu^3+^. The emission spectrum mainly presents four emission peaks, located at 597 nm, 617 nm, 658 nm, and 700 nm, respectively, under excitation wavelength of 398 nm, which correspond to excited state ^5^D_0_→^7^F_J_ (J = 1~4) transition of the Eu^3+^ ion. The main emission peak is at 617 nm (^5^D_0_→^7^F_2_), which corresponds to the red light band. The ^5^D_0_→^7^F_2_ transition is the electric dipole transition. When the Eu^3+^ ion occupies the asymmetric position, the electric dipole transition will be the main transition, which also proves the replacement of Eu^3+^ ions in the crystal structure to Ca^2+^ ions.

With the addition of Eu^3+^ ion doping amount, more luminescent centers are formed in the matrix, which increases the luminescence intensity. The emission intensity appears to reduce after it reaches the maximum at x = 0.03 (3 mol%). This is due to the interaction between the luminescent centers by increasing the Eu^3+^ concentration, the continuous migration of the excitation energy between the centers will not generate effective radiation. Therefore, 3 mol% is the best doping concentration of Eu^3+^.

### 3.3. Ca_0.97_TiO_3_: Eu^3+^_0.03_ Phosphor Synthesized by the Microwave-Assisted Sintering

Microwave-assisted sintering has many advantages, including rapid heating and energy saving. However, the Ca_0.97_TiO_3_: Eu^3+^_0.03_ phosphor cannot absorb the microwaves; thus, a small crucible with dry Ca_0.97_TiO_3_: Eu^3+^_0.03_ phosphor powder was placed in a large crucible filled with 3/4 volume of activated carbon. The activated carbon was heated using 2.45 GHz microwave radiation with a maximum power of 800 W. The precursor was heated by heat transfer from the activated carbon to prepare the Ca_0.97_TiO_3_: Eu^3+^_0.03_ phosphor. We note that the sintering atmosphere in the microwave oven might also slightly change due to the existence of activated carbon underneath the sample crucible. To record the temperature in the microwave oven, we placed a thermocouple into the crucible. The insulation inside the microwave oven is relatively poor, thus, we used an electric potential difference meter to calibrate the instantaneous temperature.

**Figure 8:**
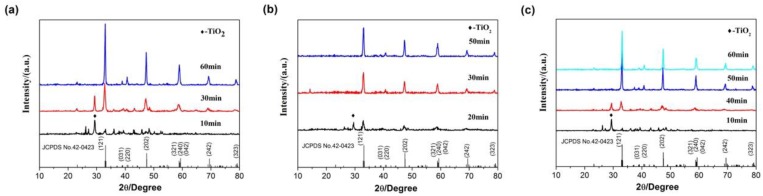
XRD patterns of the Ca_0.97_TiO_3_:Eu^3+^_0.03_ sintered at different microwave sintering power. (**a**) 800 W; (**b**) 680 W; (**c**) 528 W.

Figure 8 shows the XRD patterns of the Ca_0.97_TiO_3_:Eu^3+^_0.03_ phosphor samples sintered at different power. It can be seen in Figure 8a that the Ca_0.97_TiO_3_:Eu^3+^_0.03_ phosphor can be synthesized with microwave-assisted heating at 800 W for 30 min (at a temperature around 1100 °C) while still in the presence of TiO_2_ crystals, which indicates 30 min is not long enough for the fully crystallization of the Ca_0.97_TiO_3_:Eu^3+^_0.03_ phosphor prepared by microwave-assisted sintering at 800 W. The diffraction peaks of the samples are corresponding to the standard cards JCPDS NO.42-0423, which is, the orthorhombic CaTiO_3_ structure. From Figure 8b, we can see that the Ca_0.97_TiO_3_:Eu^3+^_0.03_ phosphor is fully crystallized at the power of 680 W for 50 min (at a temperature around 1025 °C). The results in Figure 8c is in agreement with Figure 8b, where the pure Ca_0.97_TiO_3_:Eu^3+^_0.03_ phosphor were synthesized at 528 W for 50 min (at a temperature around 950 °C). Therefore, a sintering duration of 50 min can synthesize the pure Ca_0.97_TiO_3_:Eu^3+^_0.03_ phosphor in a microwave oven at different power, where, a power of 528 W for 50 min is the most energy-efficient way.

**Figure 9:**
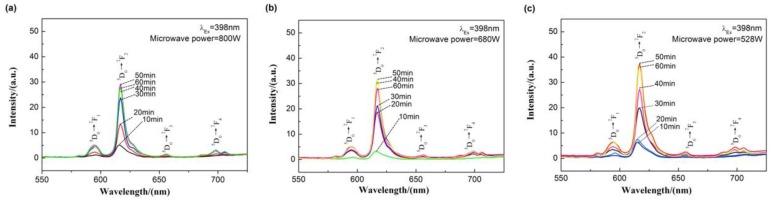
Emission spectra under an excitation wavelength of 398 nm of the Ca_0.97_TiO_3_:Eu^3+^_0.03_ prepared at different microwave sintering power. (**a**) 800 W; (**b**) 680 W; (**c**) 528 W.

Figure 9 shows the emission spectra under an excitation wavelength of 398 nm of Ca_0.97_TiO_3_:Eu^3+^_0.03_ prepared at different microwave sintering power (800 W, 680 W, and 528 W). It can be seen from Figure 9 that, under an excitation wavelength of 398 nm, the emission spectrum mainly includes four emission peaks (597 nm, 617 nm, 658 nm, 700 nm), which correspond to the ^5^D_0_→^7^F_J_ (J = 1~4) transition of Eu^3+^ ion. Moreover, with the sintering time prolonged, the emission intensity firstly increased to a peak at 50 min and then decreased. A sintering time of 60 min will not significantly increase the emission intensity, which is due to the completion of the crystallization process.

**Figure 10:**
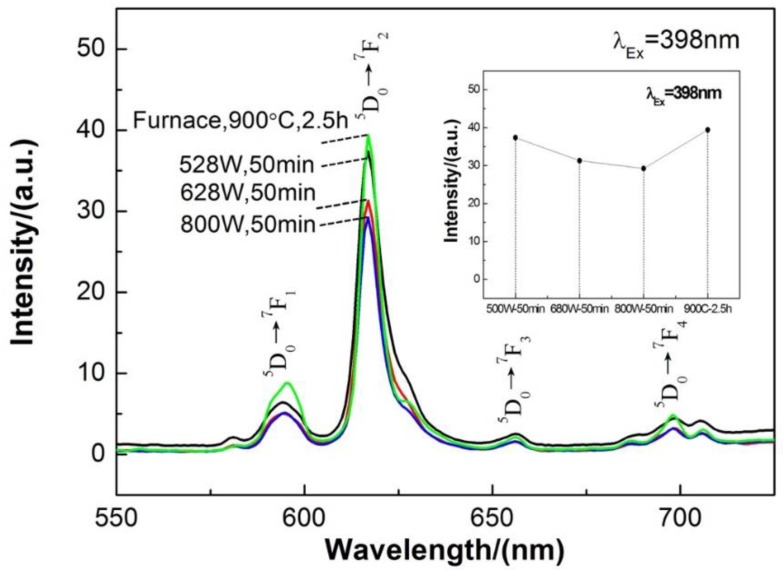
Emission spectra under an excitation wavelength of 398 nm of the Ca_0.97_TiO_3_:Eu^3+^_0.03_ synthesized by the traditional sintering process (furnace) and microwave-assisted sintering process.

Figure 10 shows the emission spectra under an excitation wavelength of 398 nm of Ca_0.97_TiO_3_:Eu^3+^_0.03_ synthesized by microwave-assisted methods and traditional furnace sintering method. A sintering power of 528 W for 50 min by microwave oven gets the similar emission intensity results as the traditional furnace sintering at 900 °C for 2.5 h. The emission intensity will significantly decrease when we further increase the power. Indeed, the microwave-assisted sintering will greatly save the energy and time for the preparation of Ca_0.97_TiO_3_:Eu^3+^_0.03_ red phosphor with good luminescent performance.

**Figure 11:**
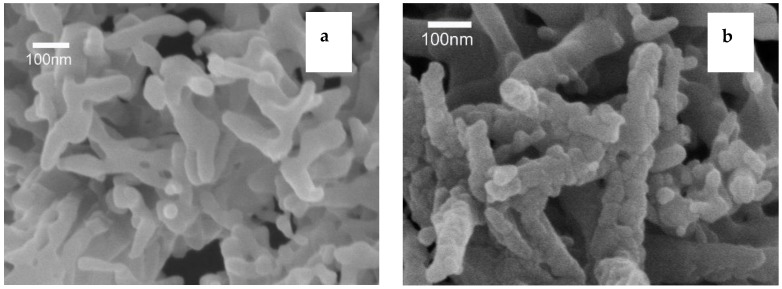
Morphology of Ca_0.97_TiO_3_:Eu^3+^_0.03_ phosphors: (**a**) synthesized by the traditional sintering processes; (**b**) synthesized by the microwave-assisted sintering process.

Figure 11 shows the morphology of Ca_0.97_TiO_3_:Eu^3+^_0.03_ phosphors (best doping) synthesized by the different synthesize methods and scanned by SEM. It can be seen from Figure 11a,b that both of the CaTiO_3_:Eu^3+^_0.03_ phosphors have a rod shape, which agrees with the orthorhombic perovskite structure from the XRD results. However, the CaTiO_3_:Eu^3+^_0.03_ phosphors synthesized by the microwave-assisted sintering process are slightly bigger while containing numerous defects on the surface compared to the ones prepared by the traditional sintering processes. The imperfection of the shape of the CaTiO_3_:Eu^3+^_0.03_ phosphors is attributed to the rapid and shorter sintering process in a microwave oven.

## 4. Conclusions

In this work, the feasibility of synthesizing Ca_1-x_TiO_3_:Eu^3+^_x_ phosphor with a good luminescent performance by combining the chemical co-precipitation method and microwave-assisted sintering was studied. First, the precursor was synthesized by the chemical co-precipitation method, which was confirmed by the FT-IR test. Then, we synthesized the Ca_1-x_TiO_3_:Eu^3+^ phosphor by traditional (furnace) sintering based on the TG-DSC analysis of the precursors. We found that the Ca_1-x_TiO_3_:Eu^3+^_x_ phosphor reached a peak emission intensity with a doping amount of 3 mol% Eu^3+^ and traditional sintering temperature of 900 °C for 2.5 h. Finally, we applied the microwave-assisted sintering with powers from 528 W to 800 W (temperature from 950–1100 °C) to prepare the Ca_0.97_TiO_3_:Eu^3+^_0.03_ phosphor and found that a sintering power of 528 W for 50 mins by microwave oven resulted in similar emission intensity results compared to traditional furnace sintering at 900 °C for 2.5 h. The new synthesized method will greatly save energy and time and is an environmentally friendly method for the preparation of Ca_0.97_TiO_3_:Eu^3+^_0.03_ red phosphor with good luminescent performance.

## Figures and Tables

**Table 1:** Lattice parameters of Ca_1-x_TiO_3_:Eu^3+^_x_ phosphors doped with various concentrations of Eu^3+^.

Phosphor	Lattice Parameters				
	X	a(Å)	b(Å)	c(Å)	v(Å^3^)
Ca_1-x_TiO_3_: Eu^3+^_x_	0.01	5.442	7.649	5.421	225.654
	0.02	5.440	7.665	5.404	225.334
	0.03	5.439	7.645	5.403	224.927
	0.04	5.419	7.662	5.406	224.460
	0.05	5.433	7.662	5.377	223.832
